# Determinants of Street Food Hygiene Practices and the Effectiveness of Interventions for Vendors and Consumers in Low- and Middle-Income Countries: Protocol for a Scoping Review

**DOI:** 10.2196/68633

**Published:** 2026-03-09

**Authors:** Abul Kamal, Tarique Md Nurul Huda, Sonjida Mesket Simi, Elizabeth D Thomas, Farhana Sultana, Jesmin Sultana, Rehnuma Haque Sarah, Soha Ishrak Rafa, Om Prasad Gautam, Peter John Winch, Mahbubur Rahman

**Affiliations:** 1 Environmental Health and WASH, Health Systems and Population Studies Division, International Centre for Diarrhoeal Disease Research, Bangladesh, 68, Shaheed Tajuddin Ahmed Sarani, Mohakhali, Dhaka, 1212, Bangladesh, +8801990857657; 2 Department of Public Health, College of Applied Medical Sciences, Qassim University, P.O. Box 6666, Qassim, Buraydah, 51452, Saudi Arabia; 3 Poverty, Gender, and Inclusion Unit, International Food Policy Research Institute, Dhaka, 1212, Bangladesh; 4 Department of International Health, Bloomberg School of Public Health, Johns Hopkins University, Baltimore, MD, United States; 5 WaterAid, Hygiene, London, United Kingdom; 6 Global Health and Migration Unit, Department of Women’s and Children’s Health, Uppsala University, Sweden

**Keywords:** food safety, hazard analysis public health, behavior change, hygiene practices, foodborne disease, street food, low- and middle-income countries, LMICs

## Abstract

**Background:**

Street food is readily available food and beverages sold by vendors, frequently situated along streets or other public spaces. Street food is largely popular because it is low cost and readily available. However, unsafe food hygiene behaviors and conditions contribute to a substantial burden of foodborne illness. There is a gap in understanding what factors determine food hygiene behavior among street food vendors and consumers in low-income countries (LICs) and low- and middle-income countries (LMICs).

**Objective:**

This review aims to identify the determinants of safe food hygiene behavior and explore the effectiveness of food hygiene interventions for vendors and consumers in LICs and LMICs.

**Methods:**

We will search the PubMed, Cochrane Library, Web of Science, ProQuest, and Scopus databases for peer-reviewed articles and Google and Google Scholar for gray literature published in the English language from inception to October 31, 2023. We will apply the World Bank 2023 definition of LICs and LMICs to include studies from these countries in this review. Two independent reviewers will search, screen, and analyze the included literature. Data will be analyzed by narrative synthesis for all objectives.

**Results:**

This protocol outlines the methodology for conducting a scoping review. The findings will identify gaps in the existing literature and map the determinants of street food hygiene, as well as the interventions implemented for street food vendors and consumers. The project received funding in October 2022 and institutional review board approval in December 2022. Data analysis is currently underway, and the first results are expected to be submitted for publication in April 2026.

**Conclusions:**

To the best of our knowledge, this will be the first scoping review to investigate the determinants of street food hygiene behaviors and the effectiveness of interventions for vendors and consumers on street food hygiene in LICs and LMICs.

## Introduction

### Background

Ready-to-eat food and beverages sold in public places or in informal settings using temporary or mobile infrastructure are generally referred to as street food [1]. Street-vended food is susceptible to chemical and microbiological contamination due to factors including vending location, raw materials and utensils used, storage and reheating practices, and the personal hygiene of street vendors [2]. Food hygiene is an important component of food safety. It includes behaviors and practices involved in the handling, preparing, storing, and serving of food to minimize the likelihood of individuals falling ill with foodborne illnesses. Street food vendors can play a role in transmitting disease while handling, preparing, serving, and storing food [2-4]. Multiple studies have found an association between contaminated street food and poor hygiene behaviors and practices, including storing food uncovered or at inappropriate temperatures, unhygienic handling of food, using poorly cleaned dishes, using a contaminated water supply, suboptimal reheating of food, and preparing and serving food in unhygienic surroundings and in the absence of handwashing and sanitation facilities [2,4-11]. Contaminated street food has been linked with foodborne disease outbreaks, including hepatitis A and cholera [12-14], and foods sold on the street are frequently contaminated with bacteria including *Salmonella* species, *Shigella* species, and *Escherichia coli*, which can pose serious health concerns to the general public [15,16]. An indicator of fecal contamination in food is *E coli,* which was found in 100% of street food samples in Nakawa, Uganda, and 43.36% of samples in Kathmandu [6,15]. In Coimbatore, Tamil Nadu, India, 70% of food samples from street vendors contained *Salmonella* species [17], and in Bangladesh, *Shigella* species was detected in 12% of food samples collected from both fixed and mobile food vendors [18]. The presence of *E coli*, *Salmonella* species, *and Shigella* species in the food may be linked to poor hand hygiene among food workers and lack of good manufacturing practices [15].

One of the leading causes of morbidity and death worldwide is foodborne illness. In 2010, the World Health Organization estimated that 600 million people experienced foodborne illness, with 420,000 related deaths [14,19]. The disease burden is higher in low- and middle-income countries (LMICs), where street food is popular due to its wide availability and low cost [1,2]. Inadequate sanitation and hygiene facilities and practices, limited surveillance, and a deficit in awareness and food hygiene education contribute to the heightened disease burden in LMICs [20,21]. After the COVID-19 pandemic, there has been a notable increase in informal businesses across many LMICs driven by economic necessity [22-25]. The pandemic significantly disrupted formal employment, prompting many individuals to seek alternative income sources within the informal sector [24,25]. At the same time, the COVID-19 pandemic may have influenced consumer behavior, with some individuals choosing to support street food vendors out of a sense of solidarity and a desire to assist those facing economic hardship [26].

Several food hygiene and safety interventions have been implemented in low-income countries (LICs) and LMICs to target street food vendors and consumers to raise awareness about the importance of street food hygiene to reduce the cases of foodborne illnesses [27-32]. However, the findings from these studies have not yet been synthesized to inform policy recommendations for LICs and LMICs. To inform effective interventions, a comprehensive understanding of the determinants of street food hygiene is needed. This requires looking beyond food hygiene knowledge and taking a closer look at the social, technological, physical, and psychosocial factors that influence the food hygiene behaviors and practices of vendors and consumers, as well as related food contamination [33,34]. Food hygiene promotion programs are likely to be most successful if they address a range of critical behavioral determinants [35]. Several studies have identified determinants of food hygiene behaviors and practices in different settings [2,4,11,36]. For instance, Birgen et al [4] found that vending places, storage facilities, and appropriate clothing are essential factors contributing to *E coli* contamination in raw chicken products vended in Nairobi city [4]. In other studies, knowledge and awareness, along with contextual factors such as settings, were also identified as important behavioral determinants [33,34].

Systematic reviews assessing the effects of education interventions and training on food safety knowledge, attitudes, and behaviors of food handlers have indicated improvements in these domains among restaurant-based vendors as a means to avoid food safety risks [37-40]. Some studies looked at ways to improve food hygiene among street food vendors in LICs and LMICs [36,41]. For instance, a study by Umar et al [36] in Nigeria revealed an enhancement in both knowledge and implementation of food hygiene practices among street food vendors following a training intervention. While several studies have assessed the impact of interventions on food hygiene practices among street food vendors, there is limited evidence on interventions targeting consumers, particularly regarding their behaviors around food handling and consumption. Currently, no systematic reviews explore the effects of food hygiene interventions on behavior and microbial outcomes on street food are registered on PROSPERO (International Prospective Register of Systematic Reviews). There is also no current scoping review on the determinants of street food hygiene in LICs and LMICs, and there are no interventions for street food vendors and consumers in LICs and LMICs to improve hygiene. Given that this area remains relatively unexplored, this review aims to synthesize evidence related to street food hygiene (concept) among vendors (population) in LICs and LMICs (context). The focus will be on the determinants of safe street food hygiene behaviors and the impact of street food hygiene interventions on street food vendors’ and consumers’ food hygiene behavior and microbiological contamination.

### Objectives

The specific objectives of this review are to synthesize existing evidence on (1) the determinants of food hygiene behavior among street food vendors, (2) explore the effects of food hygiene interventions targeting street food vendors on food hygiene behavior and the microbiological contamination of street food in LMICs and LICs, and (3) explore the effects of food hygiene interventions for consumers on safe street food consumption in LMICs and LICs.

## Methods

### Protocol Design

**Figure 1.**
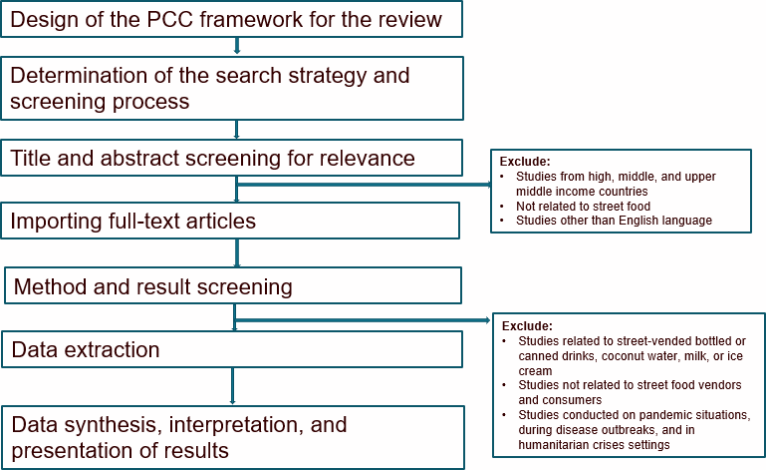
Flow diagram of the steps for conducting the scoping review. PCC: population, concept, and context.

We will follow the methodological frameworks proposed by Arksey and O'Malley [42] and Levac et al [43] for conducting a scoping review (Figure 1) [44]. This framework comprises six key phases: (1) specifying the research objectives, (2) finding relevant studies, (3) screening studies in line with predetermined criteria, (4) charting the data, (5) summarizing the findings, and (6) confirming the study results with potential stakeholders involved. Implementing this framework ensures the overall reliability of the review.

For example, it suggests involving 2 reviewers for the initial screening of titles and abstracts and piloting data extraction to maintain consistent alignment with the research objectives during the extraction process [45]. This framework helps us map available evidence on the determinants of street food hygiene and interventions related to street food hygiene among vendors, focusing on food hygiene behavior and microbiological contamination, as well as interventions targeting consumers in LICs and LMICs. It also enables us to capture diverse behavior change strategies across various informal food vending settings.

### Identification of the Research Objectives

We used an iterative process to develop the research objectives for this scoping review, including familiarization with published work on street food and subsequent discussions with the research team.

### Study Identification

#### Inclusion and Exclusion Criteria

Articles published in the English language from inception to October 31, 2023, will be considered for review. Studies will be included based on the PCC (population, concept, and context) framework. Eligible studies must address the PCC framework [46]. We will include all observational and experimental studies for objective 1. We will only include those studies that show an association between determinants and food hygiene behavior (food handling, preparation, serving, and storage) for objective 1. For objectives 2 and 3, we will include only intervention studies (experimental and quasi-experimental). Detailed information on the inclusion criteria for this review can be found in the Population and Context and the Concept sections. Studies related to street-vended bottled or canned drinks, coconut water, milk, and ice cream will be excluded.

#### Population and Context

The context for this review is low-resource settings, which include both LICs and LMICs as classified by the World Bank in 2023, comprising a total of 82 countries [47]. The population of interest will be street food vendors and consumers from LICs and LMICs. Studies related to street food vendors will be included for objectives 1 and 2, while studies related to street food consumers will be included for objective 3. We define a street food vendor as one who sells food from a temporary or mobile stall in the street or an open public location [48]. We define a street food consumer as an individual who acquires and consumes food or beverages made and sold by vendors in public areas such as streets, marketplaces, or parks, typically for immediate consumption. Articles not related to street food (eg, home cooking) will be excluded. Regarding context, studies conducted on pandemic situations, during disease outbreaks, and in humanitarian crisis settings will be excluded.

#### Concept

The core concept for this review is street food hygiene. Defining “street food hygiene” in LICs and LMICs presents particular challenges due to several context-specific factors such as inadequate infrastructure, diverse vendor food handling practices, challenges in country-specific regulations, lack of education, and socioeconomic constraints [49-52]. Street food vendors often face limited access to clean water and sanitation facilities in many LICs and LMICs, which are essential for maintaining hygiene [52]. The complexity is further compounded when there is insufficient government oversight and enforcement of food safety regulations, which allows unhygienic practices to persist unchecked [50]. To guide our review, we adopted the definition of street food hygiene from the World Health Organization and the Food and Agriculture Organization. We characterize street food hygiene as the actions undertaken by food vendors throughout the preparation process (such as handwashing with soap prior to or during food preparation, washing utensils, and maintaining specific cooking temperatures); during food handling (including preventing cross-contamination between cooked and raw foods and maintaining overall kitchen cleanliness); during serving (including handwashing with soap before serving and using clean utensils); and during food storage (ensuring storage at appropriate temperatures and reheating before consumption). These measures also encompass actions taken by consumers during the handling, consumption, and storage of purchased food [53]. The definitions of preparation, handling, serving, and storing are described in Table S4 in Multimedia Appendix 1. The definitions of determinants are described in Table S5 in Multimedia Appendix 1.

### Data Sources

The research team will use the search terms to explore a range of electronic databases for peer-reviewed and gray literature articles. Two reviewers will jointly search the PubMed, Cochrane Library, Web of Science, ProQuest, and Scopus databases for peer-reviewed articles. Google and Google Scholar will be searched for gray literature (reports, theses, and dissertations). The reviewers will do a manual search of all included articles’ reference lists to find any more pertinent papers for the review and to ensure the thoroughness of the search. We will exclude any type of review articles related to the topic; however, the reference lists within these reviews will be examined to identify relevant articles. Non–peer-reviewed book chapters will also be excluded. Peer-reviewed journal articles will be included, while unpublished theses, dissertations, and reports will be treated as gray literature and will be included. Conference proceedings will be excluded.

### Search Strategies

To find keywords for an advanced search, a preliminary search for published scientific articles on the subject of interest will be carried out. Both the PCC and Behavior-Centered Design (BCD) frameworks will be referenced for developing search strategies for all 3 objectives to ensure completeness of possible determinants that influence street food hygiene behaviors [54].

The BCD framework, which draws from multiple disciplines, including psychology and behavioral economics, will be referenced to help capture the behavioral determinants discussed in the literature in the context of street food hygiene [54]. This framework has been widely applied to help identify behavioral determinants in water, sanitation, and hygiene research [33,35]. It includes a broad set of determinants that fall into 4 main categories: environment (climate and society), settings (infrastructure and norms), brain (knowledge and motivation), and body (senses and traits). The range of determinants included in the BCD framework will facilitate the capture of the determinants of food hygiene behavior from the literature.

The search strategy will be adjusted according to the instructions of each database. Truncations will be used to broaden the search where appropriate. Synonyms and Medical Subject Headings terms will be used alongside the “OR” Boolean operator to broaden the search. The “AND” Boolean operator will combine the terms effectively to ensure relevance and prevent an influx of unrelated articles. Search terms will first be piloted in any database, downloading a few relevant articles based on the objectives. If required, we will adjust the search strategies and perform a second search to ensure we retrieve relevant articles. If we successfully identify relevant articles, we will proceed to search another database; if not, we will continue refining the search criteria until we do find the relevant articles.

To address objective 1, we developed search terms for each determinant using the BCD framework and terms related to street food hygiene behaviors. In this review, we defined behavioral determinants as those that influence behaviors positively and negatively within the behavioral context and settings such as physical, social, and biological environment; motives; barriers; social-eco-demo-cultural variability; power relations, etc. Search terms were categorized into 3 groups: terms related to street food or street food vendors (population), terms related to determinants, and terms related to food hygiene behaviors and practices (ie, patterns of behaviors; concept). For objectives 2 and 3, we used terminology associated with behavioral determinants to identify interventions that incorporated behavioral strategies grounded in a theoretical behavior change framework (concept). Additionally, we also used broader intervention-related terms. Textbox 1 presents the selection of search terms used for all 3 objectives. The complete search strategies used for the PubMed database are provided in Tables S1-S3 in Multimedia Appendix 1. A librarian will be invited to review the search strategies. The search strategy will be developed by 2 reviewers with input from a third reviewer. For content validity, search terms will be reviewed by the team members, composed of experts in the field.

Textbox 1. **Key search terms to be used to search articles.**

**Sample search terms for objective 1**

- Population-related terms (combined by “OR”): “vendor, street vended, street vendor, mobile food vendor, street market, food truck, food cart, hawker, drink, juice”
- Determinant-related terms (combined by “OR”)

  o Theory-related terms: “behavior, theory, framework, factor”

  o Determinant-related terms: “reward, trade-off, contamination, age, gender, education, skill, capacity”

- Food hygiene behavior– and contamination-related terms (combined by “OR”): “food preparation practice, reheat, temperature, storage, hazard analysis critical control point, *Salmonella, E. coli, Shigella”*

**Sample search terms for objectives 2 and 3**

- Population-related terms (combined by “OR”): “vendor, street vended, street vendor, mobile food vendor, street market, food truck, food cart, hawker, ready to drink, drink, juice, customer, buyer, consumer”
- General intervention and determinant-related terms (combined by “OR”)—intervention-related terms: “intervention, counselling, method, evaluation, video, booklet, demonstration, poster, framework, handwashing facilities, inspection”
- Food hygiene behavior—and contamination-related terms (combined by “OR”): “food preparation practice, reheat, temperature, storage, hazard analysis critical control point, *Salmonella, E. coli, Shigella”*

### Article Screening

This review will follow the Preferred Reporting Items for Systematic Reviews and Meta-Analyses extension for Scoping Reviews (PRISMA-ScR) reporting guidelines for the screening process [55]. We will go through four phases in selecting articles: (1) identification, (2) screening, (3) eligibility assessment, and (4) inclusion. The search results will be imported into EndNote (version X9; Clarivate), where duplicate articles will be removed. The updated list will then be imported into Rayyan (Rayyan Systems, Inc) online software, where additional duplicates will be identified and eliminated. To ensure consistency among reviewers, a pilot test will be conducted before the screening. The screening criteria will be refined based on pretesting with approximately 20 titles and abstracts to ensure the capture of relevant studies.

Initially, 2 reviewers will independently assess the titles and abstracts of articles using the specified inclusion and exclusion criteria. In case of any queries or disagreements between the 2 reviewers, a third reviewer will step in to address them. Subsequently, the full text of the selected articles will be reviewed by the 2 reviewers to make the final decision on inclusion. After completing the review, we will use the PRISMA (Preferred Reporting Items for Systematic Reviews and Meta-Analyses) flow diagram to report the final numbers. The reviewer will review each excluded article again after screening the title and abstract to ensure that no pertinent articles have been mistakenly excluded. Studies for which it is unclear whether the criteria will be met based on the title and abstract will undergo further discussion among the reviewers to reach a decision on inclusion. All the remaining articles at this stage will undergo a comprehensive full-text screening, where the methodology and results sections will be primarily evaluated to determine if the study’s approach aligns with the objectives of the scoping review. Any discrepancies in eligibility decisions will be resolved through discussions among the reviewers.

### Data Extraction

At least 2 reviewers will work together to extract data from the included articles according to the 3 objectives (eg, determinants of food hygiene, the effect of the intervention for vendors, and the effect of the intervention for consumers). We will also extract data on microbial contaminations including study design; location; sample size; settings of street vendors (eg, urban slum, school, and market); study duration; and intervention characteristics.

For objective 1, we will extract data related to the determinants and hygiene behaviors measured, as well as any reported associations between them. For objectives 2 and 3, we will extract the description of the intervention (eg, health education); outcome indicator (how the intervention was assessed and how microbial contamination was measured); theory, model, and framework used (if applicable); duration of the intervention; type of contact with the intervention (eg, one-to-one counseling); frequency of intervention contact; primary and secondary outcome of the intervention; and outcomes related to microbial contamination. These fields were informed by our preliminary search and refined based on the PCC and BCD frameworks. We will collect and sort key information from the selected studies in a Microsoft Excel spreadsheet following a framework developed and piloted by the team and refined using an iterative approach. Data will be extracted and charted for quantitative and qualitative descriptive analyses. Data from eligible studies will be compiled by objective in the framework in Excel.

### Data Analysis and Synthesis

Once the data extraction is finished, for objective 1, we will proceed to encode the determinants identified according to the definitions in the BCD checklist [54]. For objectives 2 and 3, we will use the Behavior Change Technique Taxonomy v1 framework by Michie et al [56] to identify and code behavior change techniques used in the included intervention studies. The Behavior Change Technique Taxonomy v1 is a framework designed to identify and categorize the smallest observable and reproducible components used in intervention studies. Two reviewers will independently carry out the coding and categorization of the results. To validate the process, a third reviewer will cross-check a random 25% of classifications. In case of any disagreements, the reviewers will discuss their coding decisions and resolve any differing opinions by providing a rationale. To address potential heterogeneity and ensure a realistic scope, we will conduct data extraction, analysis, and quality appraisal separately for each objective. For instance, for objectives 2 and 3, we will present summary tables outlining intervention characteristics, duration, type, and frequency of contact, and intervention outcomes. If a sufficient number of homogeneous intervention types are not identified, a systematic review with meta-analysis may not be feasible. Data analysis will be conducted using a narrative synthesis method in accordance with all 3 objectives [46,57]. Quantitative data from the included studies will be summarized using descriptive statistics where appropriate, while qualitative findings will be analyzed thematically. The outcomes of the interventions for objectives 2 and 3 will be synthesized separately for each objective, enabling relevant comparisons between different interventions. We will map determinants and intervention characteristics, including types and components of interventions and reported outcomes (eg, knowledge, attitude, practices, and microbiological contamination). We will highlight gaps in the existing evidence to inform future research and intervention design.

### Quality Appraisal

Gray literature will include unpublished reports, theses, and dissertations. To assess the quality of gray literature, we will use the Authority, Accuracy, Coverage, Objectivity, Date, and Significance checklist [58]. We will conduct a quality appraisal of the included studies. For objective 1, we will use the Joanna Briggs Institute critical appraisal tools according to the included study design [59]. For instance, the Joanna Briggs Institute cross-sectional checklist assesses 8 specific criteria, detailed in Table S6 in Multimedia Appendix 1. Each of the criteria will be rated as “yes” (1 point), “no” (0 points), “unclear” (0.5 points), and “not applicable (N/A).” For objectives 2 and 3, we will use a modified risk of bias (RoB) assessment tool to evaluate the potential biases across different study designs included in this review [60]. This tool was developed by the National Heart, Lung, and Blood Institute to examine controlled intervention and pre-post studies lacking a control group. The adapted RoB assessment tool comprises 14 specific criteria, detailed in Table S7 in Multimedia Appendix 1. Each of the 14 criteria will be assigned a numerical score: “yes”=1; “partially”=0.5 (where relevant); and “no,” “not applicable,” or “not reported”=0. In the context of each study, a score of 1 or “yes” will be considered as good quality, a score of 0.5 or “partially” or “unclear” will be considered as fair quality, and a score of 0 or “no” will be considered as poor quality. A study will be considered “good” when it has minimal bias, making it more likely that results are reliable and valid. A “fair” study acknowledges some bias but not enough to dismiss its findings. In contrast, a “poor” rating indicates a substantial RoB, raising concerns about the credibility of the results. The overall RoB for each study will be determined by adding up the individual scores assigned to each item. For studies under objective 1, the RoB index ranges from 0 to 8. For objectives 2 and 3, the RoB index ranges from 0 to 14. A score of 0 signifies the highest potential bias, whereas scores of 14 for objectives 2 and 3 and 8 for objective 1 represent the lowest potential bias. Two reviewers (AK and SMS) will individually assess the RoB in each study, and disagreements will be resolved with consensus between the 2 reviewers. After completing the quality assessment of the studies included for objective 1, we will present the quality ratings of the included articles alongside each determinant.

### Consultation and Dissemination

According to the suggestion of Arksey and O’Malley [42] and Levac et al [43], we will engage hygiene behavior experts as consultants throughout the review process. Consultants will provide input regarding search strategies to integrate food hygiene behavior–related terms to capture relevant studies. They will also guide the formation of data charting tables and data analysis. Through the dissemination program, we will also share the findings of this scoping review with consultants and relevant stakeholders, which will help us gain insight beyond what is reported in the literature. This involvement will be crucial in obtaining insights that may not be captured solely by existing literature. We will invite stakeholders from government, nongovernment, and international organizations who are involved with food hygiene–related programs in Bangladesh.

### Ethical Considerations

This study received ethical approval from the Ethical Review Committee of the International Centre for Diarrheal Disease Research, Bangladesh, and the approved research protocol number is PR-22143. Institutional review board Federal wide Assurance number: 00001468.

## Results

**Figure 2.**
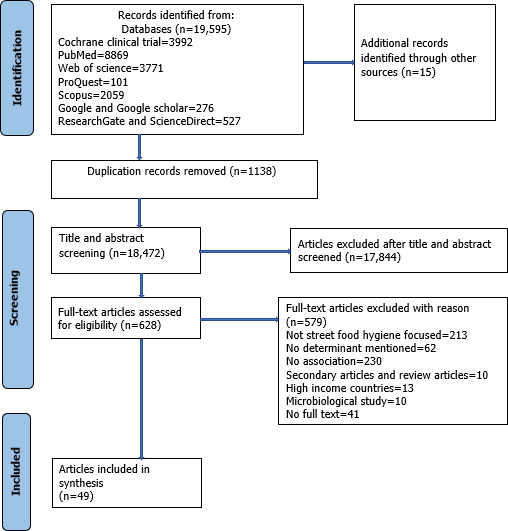
PRISMA (Preferred Reporting Items for Systematic Reviews and Meta-Analyses) flowchart highlighting the study selection process for all 3 objectives.

The project received funding in October 2022 and institutional review board approval in December 2022. We have completed the database search, title and abstract screening, and full-text review. Initially, we identified 19,595 articles, and as of October 2025, 49 (0.3%) articles have been included for synthesis (Figure 2). At present, we are conducting data analysis and quality appraisal and anticipate completing the review by April 2026. This scoping review will map the determinants of street food hygiene and the interventions implemented for street food vendors and consumers in low-resource settings. The findings will provide insights into the key factors influencing street food hygiene in LICs and LMICs and support the design of feasible, sustainable, and scalable interventions targeting both vendors and consumers.

## Discussion

The prevalence of foodborne disease is still high worldwide, especially in LICs and LMICs, where street food is popular because it is accessible and reasonably priced [61]. Despite this, synthesized evidence on behavioral determinants and effective hygiene interventions in these settings is lacking. There is an urgent need to inform context-relevant solutions, as repeated outbreaks of foodborne illness are linked to unsafe street food [2]. To help identify and organize behavioral determinants for this review, we took guidance from the BCD framework, which has been frequently used in water, sanitation, and hygiene research. The behavioral determinants outlined in the BCD framework include those related to the body (characteristics and senses); brain (executive, reactive, and motivated); environments (biological, social, and physical); and settings (stage, props, roles, routines, norms, and script) that influence individual behaviors at a given moment [54].

Several interconnected factors, such as vendor practices, environmental constraints, and consumer behavior, may influence street food hygiene [62]. This review methodology tackles a complex problem that needs in-depth investigation by considering the viewpoints of both vendors and consumers and looking at microbiological results in addition to behavior. This review will be the first evidence synthesis that examines street food hygiene determinants and interventions for vendors and consumers in LICs and LMICs [1]. It will also examine the existing evidence on the effectiveness of various interventions implemented on street food vendors and consumers, facilitating a comparison of these interventions. The understanding of the determinants of street food hygiene in LICs and LMICs identified will inform the development and evaluation of appropriate, targeted street food hygiene interventions and policies. If the evidence on determinants is found suboptimal, the gap identified and disseminated will inform researchers to conduct future studies. It will also underscore the importance of designing appropriate research studies to address any knowledge gaps identified.

## Supplementary material

10.2196/68633 Multimedia Appendix 1 It provides detailed supplementary materials supporting this scoping review protocol. It includes the complete PubMed search strategies used for objectives 1-3 (Tables S1-S3), operational definitions of food hygiene behaviors and determinants adapted from the behavior-centered design framework (Tables S4-S5), and the Joanna Briggs Institute (JBI) checklist for cross-sectional studies and the risk of bias assessment tool (Tables S6-S7).

## Abbreviations

BCD: Behavior-Centered Design
LIC: low-income country
LMIC: low- and middle-income country
PCC: population, concept, and context
PRISMA: Preferred Reporting Items for Systematic Reviews and Meta-Analyses
PRISMA-ScR: Preferred Reporting Items for Systematic Reviews and Meta-Analyses extension for Scoping Reviews
RoB: risk of bias
