# Supplementary material for: Determinants of Street Food Hygiene Practices and the Effectiveness of Interventions for Vendors and Consumers in Low- and Middle-Income Countries: Protocol for a Scoping Review
Source: JMIR Res Protoc. 2026 Mar 9;15:e68633. doi: 10.2196/68633 (PMC12978974; doi:10.2196/68633)
Supplement: Multimedia Appendix 1 [file resprot-v15-e68633-s001.docx]

**Multimedia Appendix 1 Supplementary Materials**

**Table S1:** **Objective one search strategies (PubMed):**

| **(#1)**  **Street food/ street food vendor related terms (population)** | Street OR vend* OR Vendor* OR “street vended” OR “street vendor” OR “mobile food vendor” OR Hawker OR “Street food*” OR “street sold food*” OR “street market*” OR “street meal” OR “food truck*” OR “food cart*” OR “fast food*” OR “junk food*” OR “Finger food*” OR “ready to eat food*”  **(a)** |
| --- | --- |
|  | Street OR Hawker OR vend* OR “street vended” OR “street vendor” OR “mobile vendor” OR “street sold” OR “street market*” OR drink truck* OR “food truck*” OR “food cart*” OR “ready to drink”  **(b)** |
|  | **beverage*** OR **“carbonated beverage*”** OR “**fermented beverage*”** OR “**artificially sweetened beverage*”** OR drink* OR juice* OR “raw fruit juice*” OR “fresh fruit juice*” OR “fruit juice*” OR “raw fruit*” OR “fresh Fruit*” OR “vegetable juice*” OR **“fruit and vegetable juice*” OR “Raw vegetable”**  **(c)** |
|  | **#1 a OR (b AND c)** |
| **(#2)**  **Determinants related terms (Concept)** | behav* OR theor* OR framework* OR determinant* OR factor* OR drive* OR belie* OR model OR analys* OR plan* OR barrier* OR motiv* OR facilitator* OR change* OR know* OR intend OR intent* OR influen* OR sham* OR disgust* OR comfort* OR affiliat* OR nurtur* OR status OR attract* OR lust OR love OR fear OR creat* OR curious* OR justice OR hoard* OR hunger OR hungry OR play* OR aspir* OR cue* OR trigger* OR emotion* OR **attitude*** OR habit* OR Remind OR forget* OR repetition OR repeat OR Incentive* OR prize* OR fine OR reward* OR discount* OR cost OR expens* OR award OR “self-efficac*” OR efficacy* OR trade-off* OR effort OR eas* OR busy* OR value OR benefit* OR priorit* OR expect* OR “hand hygiene education” OR Age OR Gender OR man OR men OR woman OR women OR male OR female OR wealth* OR money OR “socio-economic” OR class OR educat* OR train* OR employ* OR rural OR urban OR religio* OR cast OR faith OR personality OR trait* OR characteristic* OR “socio-demographic” OR attribute OR sense* OR dirt* OR smell* OR soft OR moistur* OR feel* OR Skill* OR capacity OR competen* OR will OR ability OR able OR commit* OR control* OR cope OR coping OR surface* OR “contaminated surface*” OR “polluted surface*” OR “surface clean*” OR environment OR setting OR clean* OR “water suppl*” OR “safe water suppl*” OR “potable water” OR “water source*” OR bucket OR tap* OR “waste disposal” OR “refuse disposal” OR “Hand washing” OR handwashing OR “Hand washing facilit*” OR “handwashing facilit*” OR “**Hand Sanitization***” OR **sanitization*** OR water OR soap OR sanitizer OR “sanitary condition” OR “Eating utensil” OR “serving Utensil*” OR “Cooking Utensil*” OR Cutlery OR lid OR “food cover*” OR crocker* OR surface* OR glove* OR hairnet* OR mask* OR Role* OR identit* OR responsibilit* OR teach* OR Norm* OR Climate OR geography OR physical OR space OR Risk OR pathogen OR outbreak OR germ OR threat OR vulnerabil* OR susceptibility* OR contaminat* OR sever* OR hazard* OR Social OR connectiv* OR network* OR friend* OR peer* OR conform* OR support* OR relationship* OR pressure* OR judge* OR observ* OR sanction* OR participat* OR Leader OR disapproval OR approval OR stigma* OR intergrat* OR enforc* OR cultur* OR regulat* OR context* OR **inspection*** OR Routine* OR script |
| **(#3)**  **Food hygiene behaviour related terms (Concept)** | “food preparation practice*” OR “food preparation” OR “manufacturing practice*” OR Preparation OR **cook*** OR Uncook* OR cookery OR **ice** OR hand* OR produce OR food OR “food stuff*” OR **“food process*”** OR “Vending surface” OR surface* OR **“food handl*”** OR handl* OR “raw food*” OR “cook food*” OR ”uncook food*” OR “Raw meat” OR serv* OR **Sick*** OR unwell OR ill OR Reheat* OR **temperature*** OR “leftover food” OR **storage*** OR “tight lid*” OR **container** OR wrapping OR “food package*” OR “cold storage” OR stor* OR “food storage*” OR **Drinking*** OR **“drinking water”** OR **water** OR Takeaway OR **“food safet*”** OR Contamination* OR “**hazard analysis critical control point*”** OR **“hazard analysis”** OR **“Critical control point*”** OR **“pest control”** OR Coli* OR Salmonella* OR Campylobact* OR Enterobact* OR Lister* OR Lyster* OR Shigella |
|  | **#1, #2 & #3 add with AND limit with English language and Human** |

**Table S2: Objective two search strategies (PubMed):**

| **(#1)**  **Street food/ street food vendor related terms (population)** | Street OR vend* OR Vendor* OR “street vended” OR “street vendor” OR “mobile food vendor” OR Hawker OR “Street food*” OR “street sold food*” OR “street market*” OR “street meal” OR “food truck*” OR “food cart*” OR “fast food*” OR “junk food*” OR “Finger food*” OR “ready to eat food*”  **(a)** |
| --- | --- |
|  | Street OR Hawker OR vend* OR “street vended” OR “street vendor” OR “mobile vendor” OR “street sold” OR “street market*” OR drink truck* OR “food truck*” OR “food cart*” OR “ready to drink”  **(b)** |
|  | **beverage*** OR **“carbonated beverage*”** OR “**fermented beverage*”** OR “**artificially sweetened beverage*”** OR drink* OR juice* OR “raw fruit juice*” OR “fresh fruit juice*” OR “fruit juice*” OR “raw fruit*” OR “fresh Fruit*” OR “vegetable juice*” OR **“fruit and vegetable juice*” OR “Raw vegetable”**  **(c)** |
|  | **#1 a OR (b AND c)** |
| **(#2)**  **General interventions & determinants related terms (Concept)** | Intervention* OR counsel* OR Method OR Evaluation OR video* OR booklet* OR demonstration* OR poster*  **(a)** |
|  | behav* OR theor* OR framework* OR determinant* OR factor* OR drive* OR belie* OR model OR analys* OR plan* OR barrier* OR motiv* OR facilitator* OR change* OR know* OR intend OR intent* OR influen* OR sham* OR disgust* OR comfort* OR affiliat* OR nurtur* OR status OR attract* OR lust OR love OR fear OR creat* OR curious* OR justice OR hoard* OR hunger OR hungry OR play* OR aspir* OR cue* OR trigger* OR emotion* OR **attitude*** OR habit* OR Remind OR forget* OR repetition OR repeat OR Incentive* OR prize* OR fine OR reward* OR discount* OR cost OR expens* OR award OR “self-efficac*” OR efficacy* OR trade-off* OR effort OR eas* OR busy* OR value OR benefit* OR priorit* OR expect* OR “hand hygiene education” OR Age OR Gender OR man OR men OR woman OR women OR male OR female OR wealth* OR money OR socio-economic OR class OR educat* OR train* OR employ* OR rural OR urban OR religio* OR cast OR faith OR personality OR trait* OR characteristic* OR “socio-demographic” OR attribute OR sense* OR dirt* OR smell* OR soft OR moistur* OR feel* OR Skill* OR capacity OR competen* OR will OR ability OR able OR commit* OR control* OR cope OR coping OR surface* OR “contaminated surface*” OR “polluted surface*” OR “surface clean*” OR environment OR setting OR clean* OR “water suppl*” OR “safe water suppl*” OR “potable water” OR “water source*” OR bucket OR tap* OR “waste disposal” OR “refuse disposal” OR “Hand washing” OR handwashing OR “Hand washing facilit*” OR “handwashing facilit*” OR “**Hand Sanitization***” OR **sanitization*** OR water OR soap OR sanitizer OR “sanitary condition” OR “Eating utensil” OR “serving Utensil*” OR “Cooking Utensil*” OR Cutlery OR lid OR “food cover*” OR crocker* OR surface* OR glove* OR hairnet* OR mask* OR Role* OR identit* OR responsibilit* OR teach* OR Norm* OR Climate OR geography OR physical OR space OR Risk OR pathogen OR outbreak OR germ OR threat OR vulnerabil* OR susceptibility* OR contaminat* OR sever* OR hazard* OR Social OR connectiv* OR network* OR friend* OR peer* OR conform* OR support* OR relationship* OR pressure* OR judge* OR observ* OR sanction* OR participat* OR Leader OR disapproval OR approval OR stigma* OR intergrat* OR enforc* OR cultur* OR regulat* OR context* OR **inspection*** OR Routine* OR script  **(b)** |
|  | **#2 a OR b** |
| **(#3)**  **Food hygiene behaviour related terms (Concept)** | “food preparation practice*” OR “food preparation” OR “manufacturing practice*” OR Preparation OR **cook*** OR Uncook* OR cookery OR **ice** OR hand* OR produce OR food OR “food stuff*” OR **“food process*”** OR “Vending surface” OR surface* OR **“food handl*”** OR handl* OR “raw food*” OR “cook food*” OR ”uncook food*” OR “Raw meat” OR serv* OR **Sick*** OR unwell OR ill OR Reheat* OR **temperature*** OR “leftover food” OR **storage*** OR “tight lid*” OR **container** OR wrapping OR “food package*” OR “cold storage” OR stor* OR “food storage*” OR **Drinking*** OR **“drinking water”** OR **water** OR Takeaway OR **“food safet*”** OR Contamination* OR “**hazard analysis critical control point*”** OR **“hazard analysis”** OR **“Critical control point*”** OR **“pest control”** OR Coli* OR Salmonella* OR Campylobact* OR Enterobact* OR Lister* OR Lyster* OR Shigella |
|  | **#1, #2 & #3 add with AND limit with English language and Human** |

**Table S3: Objective three search strategies (PubMed)**

| **(#1)**  **Street food/ street food vendor related terms (population)** | Street OR vend* OR Vendor* OR “street vended” OR “street vendor” OR “mobile food vendor” OR Hawker OR “Street food*” OR “street sold food*” OR “street market*” OR “street meal” OR “food truck*” OR “food cart*” OR “fast food*” OR “junk food*” OR “Finger food*” OR “ready to eat food*”  **(a)** |
| --- | --- |
|  | Street OR Hawker OR vend* OR “street vended” OR “street vendor” OR “mobile vendor” OR “street sold” OR “street market*” OR “drink truck*” OR “food truck*” OR “food cart*” OR “ready to drink”  **(b)** |
|  | **beverage*** OR **“carbonated beverage*”** OR “**fermented beverage*”** OR “**artificially sweetened beverage*”** OR drink* OR juice* OR “raw fruit juice*” OR “fresh fruit juice*” OR “fruit juice*” OR “raw fruit*” OR “fresh Fruit*” OR “vegetable juice*” OR **“fruit and vegetable juice*” OR “Raw vegetable”**  **(c)** |
|  | customer OR buyer OR purchaser OR public OR client* OR patron* OR shopper* OR vendee OR consumer* OR consume*  **(d)** |
|  | **#1 a OR (b AND c) AND d** |
| **(#2)**  **General interventions & determinants related terms (Concept)** | Intervention* OR counsel* OR Method OR Evaluation OR video* OR booklet* OR demonstration* OR poster*  **(a)** |
|  | behav* OR theor* OR framework* OR determinant* OR factor* OR drive* OR belie* OR model OR analys* OR plan* OR barrier* OR motiv* OR facilitator* OR change* OR know* OR intend OR intent* OR influen* OR sham* OR disgust* OR comfort* OR affiliat* OR nurtur* OR status OR attract* OR lust OR love OR fear OR creat* OR curious* OR justice OR hoard* OR hunger OR hungry OR play* OR aspir* OR cue* OR trigger* OR emotion* OR **attitude*** OR habit* OR Remind OR forget* OR repetition OR repeat OR Incentive* OR prize* OR fine OR reward* OR discount* OR cost OR expens* OR award OR “self-efficac*” OR efficacy* OR trade-off* OR effort OR eas* OR busy* OR value OR benefit* OR priorit* OR expect* OR “hand hygiene education” OR Age OR Gender OR man OR men OR woman OR women OR male OR female OR wealth* OR money OR “socio-economic” OR class OR educat* OR train* OR employ* OR rural OR urban OR religio* OR cast OR faith OR personality OR trait* OR characteristic* OR “socio-demographic” OR attribute OR sense* OR dirt* OR smell* OR soft OR moistur* OR feel* OR Skill* OR capacity OR competen* OR will OR ability OR able OR commit* OR control* OR cope OR coping OR surface* OR “contaminated surface*” OR “polluted surface*” OR “surface clean*” OR environment OR setting OR clean* OR “water suppl*” OR “safe water suppl*” OR “potable water” OR “water source*” OR bucket OR tap* OR “waste disposal” OR “refuse disposal” OR “Hand washing” OR handwashing OR “Hand washing facilit*” OR “handwashing facilit*” OR “**Hand Sanitization***” OR **sanitization*** OR water OR soap OR sanitizer OR “sanitary condition” OR “Eating utensil” OR “serving Utensil*” OR “Cooking Utensil*” OR Cutlery OR lid OR “food cover*” OR crocker* OR surface* OR glove* OR hairnet* OR mask* OR Role* OR identit* OR responsibilit* OR teach* OR Norm* OR Climate OR geography OR physical OR space OR Risk OR pathogen OR outbreak OR germ OR threat OR vulnerabil* OR susceptibility* OR contaminat* OR sever* OR hazard* OR Social OR connectiv* OR network* OR friend* OR peer* OR conform* OR support* OR relationship* OR pressure* OR judge* OR observ* OR sanction* OR participat* OR Leader OR disapproval OR approval OR stigma* OR intergrat* OR enforc* OR cultur* OR regulat* OR context* OR **inspection*** OR Routine* OR script  **(b)** |
|  | **#2 a OR b** |
| **(#3)**  **Food hygiene behaviour related terms (Concept)** | “food preparation practice*” OR “food preparation” OR “manufacturing practice*” OR Preparation OR **cook*** OR Uncook* OR cookery OR **ice** OR hand* OR produce OR food OR “food stuff*” OR **“food process*”** OR “Vending surface” OR surface* OR **“food handl*”** OR handl* OR “raw food*” OR “cook food*” OR ”uncook food*” OR “Raw meat” OR serv* OR **Sick*** OR unwell OR ill OR Reheat* OR **temperature*** OR “leftover food” OR **storage*** OR “tight lid*” OR **container** OR wrapping OR “food package*” OR “cold storage” OR stor* OR “food storage*” OR **Drinking*** OR **“drinking water”** OR **water** OR Takeaway OR **“food safet*”** OR Contamination* OR “**hazard analysis critical control point*”** OR **“hazard analysis”** OR **“Critical control point*”** OR **“pest control”** OR Coli* OR Salmonella* OR Campylobact* OR Enterobact* OR Lister* OR Lyster* OR Shigella |
|  | **#1, #2 & #3 add with AND limit with English language and Human** |

**Table S4: Definition of the food hygiene behaviour**

| **Behaviour** | **Definition** |
| --- | --- |
| Preparation | Behaviours related to using only potable water for street food cooking and making ice for street food, never reuse water used for washing utensils, food or hands, washing hands with soap and clean water by street food vendors, using clean cloth/ hand gloves/ head cap by street food vendors, keeping surfaces and vending units clean by street food vendors, having a proper waste disposal facility are listed as preparation. |
| Handling | Behaviours related to separating raw and cooked food (specially animal food), separating raw meat and raw vegetables, minimizing the time between food preparation and food serving, washing hands with clean water between handling money and food, street vendors should not handle/ prepare food if s/he is sick, covering food with tight lid all the time to protect from dust and touch of consumers, cooking food thoroughly at minimum 70⁰c temperature by street food vendors are termed as handling |
| Serving | Behaviours related to serving in a clean utensil, reheating leftover foods at minimum 70⁰c temperature, never store reheated leftover food until next day (if there is no cold storage) are termed as serving |
| Storing | Behaviours related to food storing at hot temperature (above 60⁰c) or cold temperature (less than 5⁰c), storing cooked food in a clean utensil with tight lid, storing cooked & uncooked food separately, storing drinking water fully covered all the time, not touching drinking water with clean utensil/ glass, prepared foods cannot be stored in room temperature after 4 hours (because microbiological contamination starts) are listed as storing. |

**Table S5: Definition of determinants adapted from BCD framework**

| **Determinant** | | **Definition** |
| --- | --- | --- |
| Brain | Executive Brain | The extent to which knowledge, attitude and awareness of preparation, handling, serving and storing of street food vendors and its benefits affects the intentions and plans, and eventually performance of the street food hygiene behaviour |
|  | Motivated Brain | The goal-related drivers of street food hygiene behaviour. Motives or rewards for preparation, handling, serving and storing can include (but is not limited to) disgust (the desire to avoid cues to sources of infection), affiliation (the desire to fit in with others) |
|  | Reactive Brain | The extent to which preparation, handling, serving and storing of street food vendors can be automatically triggered based on past experience and repetition |
| Body | Characteristics | Socio-demographic characteristics that may affect preparation, handling, serving and storing of street food, including gender, wealth, age, education and employment etc. |
|  | Senses | The sensory perceptions that may cue preparation, handling, serving and storing street food hygiene behaviours |
| Behaviour settings | Stage | The design and set up of the specific physical spaces where street food hygiene behaviour takes place. |
|  | Props | The material/ objects used to accomplish the behaviours of street food hygiene e.g. apron, head cap, water etc. |
|  | Roles | The ways in which an individual's role, identity or responsibilities influence their street food hygiene practices, e.g. street vendor, customer etc. |
|  | Routine | The sequence of behaviours regularly performed in association with street food hygiene, e.g. asking street vendor to give food menu, ordering food, standing or sitting down in a place etc. |
|  | Norms | The extent to which an individual's street food hygiene practice is influenced by their perception of normative setting specific rules. This includes an individual's perception of whether street food hygiene is commonly practiced in their community (descriptive norm); whether handwashing is part of their role and their normal behaviour (personal norm); whether street food hygiene is socially approved of (injunctive norm); and whether street food hygiene is practiced by their ‘valued others’ (subjective norm) |
|  | Script | An individual’s knowledge of a routine refers to a set of mental instructions about how to behave in a particular behaviour setting, to play a role, e.g. choose a food cart, order food, eat and pay to the street vendor |
| Environment | Physical environment | Factors in the physical objects, infrastructures and macrostructures including water supply systems, waste disposal facilities and handwashing facilities |
|  | Biological Environment | Factors associated with an individual's interaction within their biological environment |
|  | Social Environment | The structure of an individual's social environment, including how they interact with it and perceive themselves within it. Moreover, formal institutions with rules/ regulations/ policies for converting roles into position and for excluding/ admitting individuals into specific roles, which thereby become personalized |

**Table S6: JBI checklist for cross sectional studies**

| **ITEMS FOR CRITICAL APPRAISAL** |
| --- |
| 1. Were the criteria for inclusion in the sample clearly defined? |
| 1. Were the study subjects and the setting described in detail? |
| 1. Was the exposure measured in a valid and reliable way? |
| 1. Were objective, standard criteria used for measurement of the condition? |
| 1. Were confounding factors identified? |
| 1. Were strategies to deal with confounding factors stated? |
| 1. Were the outcomes measured in a valid and reliable way? |
| 1. Was appropriate statistical analysis used? |

**Table S7: Risk of Bias Tool**

| **#** | **RISK OF BIAS ITEMS** |
| --- | --- |
| 1 | Were the research questions or objectives in this paper clearly stated and appropriate? |
| 2 | Was the study population clearly specified and defined? |
| 3 | Was a sample size justification, power description or variance and effect estimates provided? |
| 4 | Were the intervention/s clearly described and delivered consistently against the study population? |
| 5 | Were the outcome measures pre-specified, clearly defined, valid, reliable, and assessed consistently across all study participants? |
| 6 | Was the outcome observed rather than self-reported? (only 0 or 1 possible) |
| 7 | Were the measures of outcome assessors blinded to the exposures/interventions of participants? |
| 8 | Was loss-to-follow up after baseline 20% or less? |
| 9 | Were those lost to follow-up accounted for in the analysis? |
| 10 | Were key confounding variables measured and adjusted statistically for their impact on the relationship between exposure(s) and outcome(s)? |
| 11 | Does the study have a control group? (only 0 or 1 possible) |
| 12 | Was the study randomised? (score 0 if there is no control group) |
| 13 | Were the groups similar at baseline on important characteristics that could affect outcomes? (score 0 if ‘no’ or if there is no control group) |
| 14 | Was the allocation sequence concealed? (score 0 if there is no control group) |
